# Importance of biopsy site selection for peritoneal regression grading score (PRGS) in peritoneal metastasis treated with repeated pressurized intraperitoneal aerosol chemotherapy (PIPAC)

**DOI:** 10.1515/pp-2022-0108

**Published:** 2022-05-30

**Authors:** Mojib Fallah, Sönke Detlefsen, Alan P. Ainsworth, Claus W. Fristrup, Michael B. Mortensen, Per Pfeiffer, Line S. Tarpgaard, Martin Graversen

**Affiliations:** Odense PIPAC Center, Odense University Hospital, Odense, Denmark; Upper GI and HPB Section, Department of Surgery, Odense University Hospital, Odense, Denmark; Department of Pathology, Odense University Hospital, Odense, Denmark; Department of Clinical Research, Faculty of Health Sciences, University of Southern Denmark, Odense, Denmark; Department of Oncology, Odense University Hospital, Odense, Denmark; Open Patient data Explorative Network (OPEN), Odense University Hospital, Region of Southern Denmark, Odense, Denmark

**Keywords:** biopsy, clips marked, peritoneal metastasis, Peritoneal Regression Grading Score (PRGS), Pressurized IntraPeritoneal Aerosol chemotherapy (PIPAC)

## Abstract

**Objectives:**

The four-tiered peritoneal regression grading score (PRGS) is used for histological response evaluation in patients with peritoneal metastasis (PM) treated with pressurized intraperitoneal aerosol chemotherapy (PIPAC). Four quadrant biopsies (QBs) from the parietal peritoneum should be assessed by PRGS, but consensus on biopsy site strategy for follow-up biopsies during repeated PIPACs is lacking. We aimed to evaluate whether there is a difference between PRGS in QBs from clips marked PM (QB-CM) compared to biopsies from PM with the visually most malignant features (worst biopsy, WB).

**Methods:**

Prospective, descriptive study. During the first PIPAC, index QBs sites were marked with metal clips. During the second PIPAC, an independent surgical oncologist selected biopsy site for WB and biopsies were taken from QB-CM and WB. One blinded pathologist evaluated all biopsies according to PRGS. From each biopsy, three step sections were stained H&E, followed by an immunostained section, and another three step sections stained H&E.

**Results:**

Thirty-four patients were included from March 2020 to May 2021. Median age 64 years. Maximum mean PRGS in QB-CM at PIPAC 1 was 3.3 (SD 1.2). Maximum mean PRGS in QB-CM at PIPAC 2 was 2.6 (SD 1.2), whereas mean PRGS in WB at PIPAC 2 was 2.4 (SD 1.3). At PIPAC 2, there was agreement between maximum PRGS from QB-CM and PRGS from WB in 21 patients. Maximum PRGS from QB-CM was higher in nine and lower in four patients, compared to PRGS from WB.

**Conclusions:**

Biopsies from QB-CM did not overestimate treatment response compared to biopsies from WB.

## Introduction

Pressurized intraperitoneal aerosol chemotherapy (PIPAC) is a laparoscopy-controlled local treatment for patients with peritoneal metastasis (PM) [1]. Studies have shown that PIPAC is feasible, safe, able to stabilize or improve quality of life, and can induce an objectively measurable reduction in disease burden of PM [2], [3], [4], [5]. Still, there are no randomized controlled trials, and the optimal number of PIPAC treatments in each patient is not known. Therefore, it is pivotal to be able to evaluate treatment response after each PIPAC procedure.

Treatment response in oncological studies is usually evaluated using the response evaluation criteria in solid tumors (RECIST), but PM can rarely serve as measurable targets [6]. Therefore, therapeutic response remains difficult to evaluate, whether estimated by surgeons or by abdominal imagining [7], [8], [9]. Several histological response classifications have been introduced for gastrointestinal cancers, for example the tumor regression grade in colorectal liver metastasis, which is a validated prognostic factor [10], [11], [12], [13]. In 2016, a group of European pathologists proposed the four-tiered peritoneal regression grading score (PRGS) for histological assessment of therapeutic response in PM [14]. The PRGS is widely adopted by centers performing PIPAC, and has a substantial inter-observer, and almost-perfect intra-observer variability [15]. Immunohistochemistry (IHC) can improve inter-observer variability of PRGS, particularly in less experienced observers [16]. The maximum PRGS, combined with peritoneal cytology, had prognostic value in a recent study on PIPAC treatment of PM [17]. The proposal article of PRGS recommended that four quadrant biopsies (QBs) from the parietal peritoneum should be obtained from suspect localizations, but consensus regarding the biopsy site strategy for follow-up biopsies in patients undergoing repeated PIPAC is currently lacking [14]. Some centers take biopsies from the same PM elements prior to each PIPAC, and these elements are marked by metal clips [18, 19]. Other centers take biopsies from the elements with visually most malignant features prior to each PIPAC [20, 21].

Theoretically, repeated biopsies from the same PM elements could lead to overestimated histological regression due to surgically induced fibrosis and scar tissue. Based on biopsies from the second PIPAC, the aim of this study was to compare the maximum PRGS in biopsies from clips marked PM (QB-CM) to biopsies from PM with the visually most malignant features (worst biopsy, WB).

## Materials and methods

This study was conducted as an amendment to the ongoing PIPAC-OPC2 study, which is a GCP-monitored prospective phase II study of patients with radiological, cytological or histological proven PM from gastrointestinal, ovarian, or primary peritoneal cancer [18]. Patients were discussed at a dedicated PIPAC multidisciplinary tumor conference before inclusion, and were only eligible if they had a minimum of two PIPAC procedures.

Adults with an Eastern Cooperative Oncology Group (ECOG) performance status of 0–1, non-obstructed gastrointestinal tract, and a maximum of one extraperitoneal metastasis were included. Women had to be postmenopausal, or use approved contraceptives.

Patients with a history of allergic reactions to doxorubicin or platinum, renal impairment (GFR < 40 mL/min), myocardial insufficiency (NYHA class > 2), impaired liver function (bilirubin > 1.5 upper normal limit) or inadequate hematological function (ANC <1.5 × 10 ^9^/L or plates <100 × 10^9^/L) were excluded.

The PIPAC procedure has been described previously [3, 18]. PIPAC was performed with oxaliplatin (92 mg/m^2^ body surface in 150 mL dextrose) in patients with PM of colorectal or appendiceal origin. A combination of doxorubicin (1.5 mg/m^2^ in 50 mL NaCl 0.9%) and cisplatin (7.5 mg/m^2^ in 150 mL NaCl 0.9%) were used in patients with PM of non-colorectal origin.

### Biopsy strategy

Peritoneal punch biopsies were taken from all four quadrants (if possible) with a biopsy forceps during the first PIPAC procedure, and small metal clips were used to mark the biopsy sites. QBs were subsequently taken from these locations (QB-CM). During the second PIPAC procedure, an independent expert in surgical oncology defined the PM with laparoscopically most malignant features, and one biopsy was taken from this location (WB). The QB-CM was defined within the distance of two open jaws of the biopsy forceps. Overlap between QB-CM and WB was accepted.

### Specimen evaluation

The biopsies were evaluated by one pathologist with an interest in peritoneal malignancies. The pathologist was informed of the primary tumor origin, but blinded regarding the origin of the biopsies (QB-CM vs. WB PM). The biopsies were fixed in formalin and embedded in paraffin. From the included biopsies, three-step sections were stained with H&E, and one section was IHC stained for EpCAM, as described previously [16]. For EpCAM IHC, a mouse monoclonal antibody (clone BS14 [Nordic BioSite ApS], 1:600 [20 min at 32 °C], heat-induced antigen retrieval [HIER] using target retrieval solution-high [pH 9] for 30 min at 97 °C, Omnis platform, Envision detection) was used. For biopsies with malignant mesothelioma, IHC staining for CKAE1/AE3 was used (mouse monoclonal antibody, clone AE1+AE3 [Dako], 1:100 [24 min at 36 °C], HIER using cell conditioning solution 1 [pH 8.5, Ventana Medical Systems] for 32 min at 100 °C, Benchmark Ultra platform).

Microscopically, biopsies were evaluated according to PRGS [14]. The PRGS defines four categories based on the presence of residual tumor cells, and the extent of regressive features. Major histological features of regression are regressive fibrosis, inflammation, elastosis, acellular mucin pools (in primaries other than low-grade appendiceal mucinous neoplasm), ischemic necrosis, accumulation of macrophages/multinucleated giant cells, and granuloma formation. The detailed definition of PRGS has been described previously [14, 15]. In short, PRGS 1 corresponds to a complete regression with the absence of tumor. PRGS 2 is a major histological response with regressive features predominant over residual tumor. PRGS 3 is a minor histological response with predominance of residual tumor over regressive features. PRGS 4 is used when there is no histological response [14]. According to the original article, both mean and maximum PRGS value was reported [14].

### Statistics

Values are given as means, medians or percentages where appropriate. Comparisons were performed using a Fishers exact test, p-values are two tailed, and a p-value of 0.05 is considered statistically significant. The statistical software Stata, version 16 (Stata Corp, USA) was used for statistical analyses.

### Ethics and trial registration

The PIPAC-OPC2 study is GCP monitored and has been approved by the Scientific Ethical Committees for Southern Denmark (IRB S-20160100), and the Danish Medicines Agency (code No. 2016083464, EudraCT number 2016-003394-18). Oral and written informed consent from participants was obligatory.

## Results

A total of 42 patients were included in the PIPAC-OPC2 study from March 2020 to May 2021 (Table 1). Of these, 34 patients had two PIPACs, and were eligible for evaluation of biopsy strategy in the present study. Thirty-two patients (94.1%) were treated with palliative systemic chemotherapy prior to PIPAC, and 21 (61.8%) were treated with a combination of systemic chemotherapy and PIPAC (bidirectional treatment).

**Table 1:** Baseline characteristics of patients with peritoneal metastasis treated with pressurized intraperitoneal aerosol chemotherapy.

Variable	Value, n (%)
Number of patients	34
Age, median years (range)	64 (47–79)
Sex (male/female)	17/17
ECOG performance status 0	23
ECOG performance status 1	11
Previous palliative systemic chemotherapy	32 (94.1)
One-line systemic chemotherapy	24 (70.6)
Two-lines systemic chemotherapy	4 (11.8)
Three-lines systemic chemotherapy	4 (11.8)
Bidirectional treatment	21 (61.8)
Primary tumor origin	
Gastric adenocarcinoma	12 (35.3)
Pancreatic adenocarcinoma	6 (17.6)
Colorectal adenocarcinoma	6 (17.6)
Appendix^a^	4 (11.8)
Bile ducts, adenocarcinoma	3 (8.8)
Small bowel	1 (2.9)
Malignant peritoneal mesothelioma	1 (2.9)
Ovaries	1 (2.9)
Primary tumor *in situ*	18 (52.9)
Extraperitoneal metastasis	4 (11.8)
Index PCI score, mean (SD)	12.7 (9.2)

ECOG, Eastern Cooperative Oncology Group; PCI, Peritoneal Cancer Index; SD, standard deviation. ^a^Appendix mucinous adenocarcinoma (grade 2), Appendix mixed adenoneuroendocrine carcinoma, Appendix signet ring cell adenocarcinoma.

**Figure 1:**
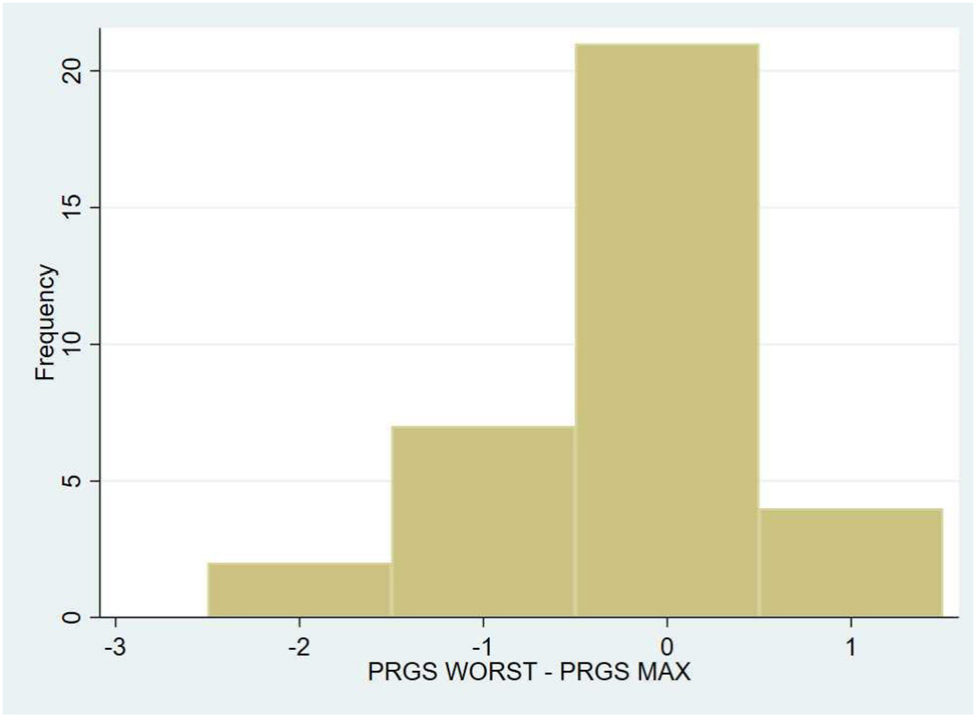
Difference in maximal peritoneal regression grading score (PRGS) between clips marked quadrant biopsies and the peritoneal metastasis with visually most malignant features (WB) at the second pressurized intraperitoneal aerosol chemotherapy procedure.

**Figure 2:**
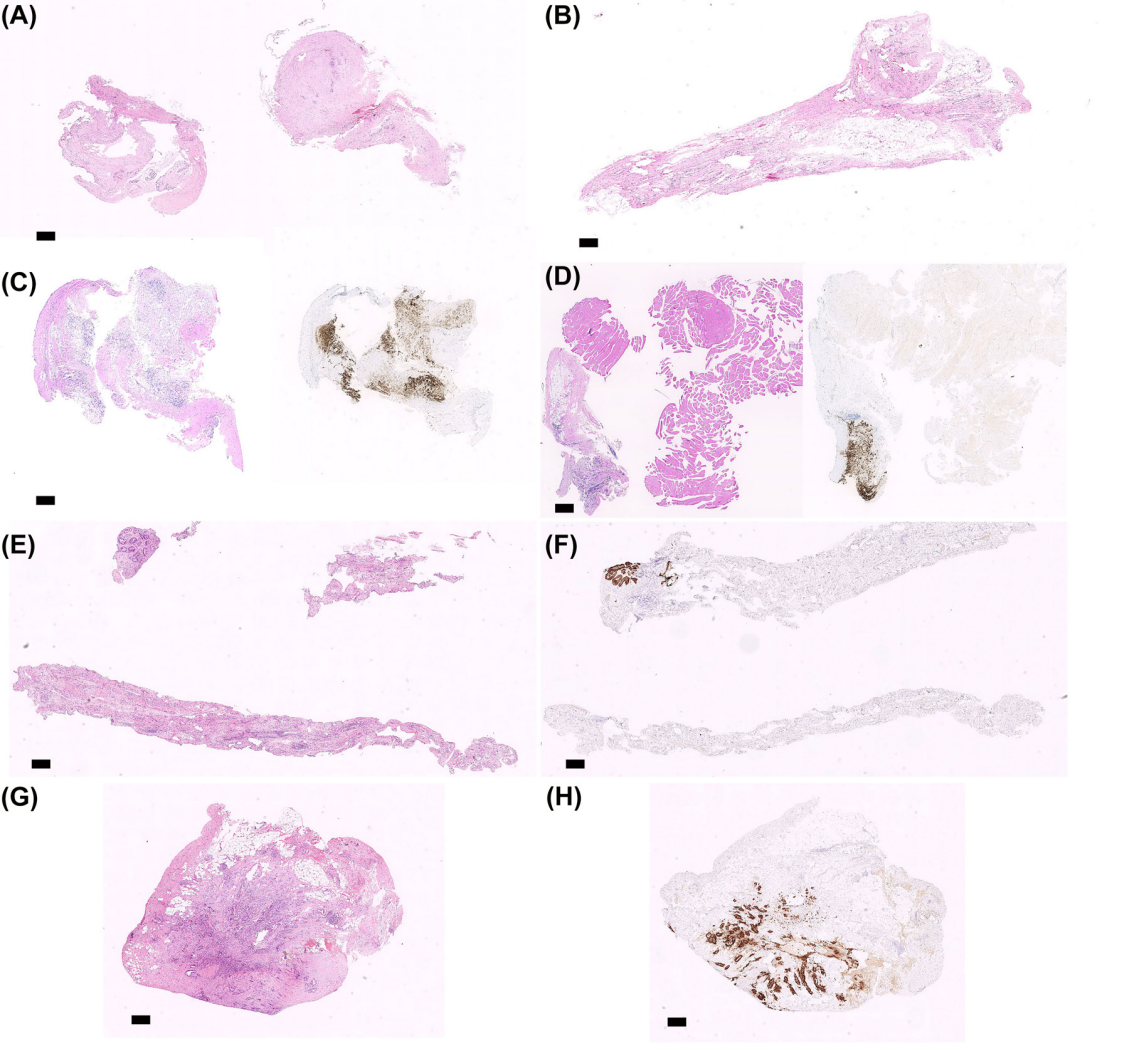
Microscopy of peritoneal biopsies taken after one pressurized intraperitoneal aerosol chemotherapy (PIPAC) treatment of peritoneal metastasis (PM). The clips marked quadrant biopsy (QB-CM) showing the maximum peritoneal regression grading score (PRGS) is shown, compared to biopsies taken from the visually most malignant features (worst biopsy, WB). (A and B) QB-CM and WB from a patient with PM from gastric cancer, both showing complete histological response (PRGS 1) (H&E). (C and D) WB and QB-CM from a patient with PM from colorectal cancer (left: H&E, right: EpCAM immunostaining). WB (C) shows a higher PRGS (score 4) compared to QB-CM (D) (score 3). Note that the majority of the QB-CM (C) consists of normal striated muscle and is therefore excluded from the PRGS assessment. (E and F) WB and (G and H) QG-CM from a patient with PM from pancreatic cancer (E and G: H&E, F and H: EpCAM immunostaining). WB (E and F) shows a lower PRGS (score 2) compared to QB-CM (G and H) (score 3). Scale bar: 0.5 mm.

Based on QB-CMs from the first PIPAC procedure, the mean and maximum PRGS was 2.9 (SD 1.0) and 3.3 (SD 1.2). The mean and maximum PRGS in QB-CM from the second PIPAC were 2.1 (SD 0.9) and 2.6 (SD 1.2), respectively, whereas the PRGS from WB was 2.4 (SD 1.3) (Table 2). There was agreement between PRGS maximum from QB-CM and WB in 21 patients. PRGS maximum from QB-CM was higher in nine patients and lower in four patients compared to PRGS WB (p < 0.05) (Figures 1 and 2).

**Table 2:** Peritoneal regression grading score (PRGS) of patients with peritoneal metastasis treated with pressurized intraperitoneal aerosol chemotherapy (PIPAC).

Tumor origin (n)	Mean PRGS (QB-CM) PIPAC 1 (SD)	Mean PRGS (QB-CM) PIPAC 2 (SD)	Maximum PRGS (QB-CM) PIPAC 1 (SD)	Maximum PRGS (QB-CM) PIPAC 2 (SD)	PRGS worst (WB) PIPAC 2 (SD)
Gastric adenocarcinoma (12)	2.4 (1.7)	1.9 (1.0)	2.7 (1.4)	2.4 (1.5)	2.6 (1.5)
Pancreatic adenocarcinoma (6)	3.3 (0.4)	2.2 (0.7)	3.8 (0.4)	3.0 (0.6)	2.7 (0.8)
Colorectal adenocarcinoma (6)	2.7 (1.4)	1.7 (0.8)	3.0 (1.5)	2.2 (1.3)	2.3 (1.5)
Bile duct, adenocarcinoma (3)	3.4 (0.4)	3.2 (0.8)	4.0 (0.0)	3.7 (0.7)	3.3 (0.6)
Appendix mucinous adenocarcinoma (grade 2) (2)	3.2 (0.7)	2.3 (1.1)	4.0 (0.0)	2.5 (0.7)	2.0 (1.4)
Appendix mixed adenoneuroendocrine carcinoma (1)	3.7	2.0	4.0	3.0	1.0
Appendix signet ring cell adenocarcinoma (1)	3.8	1.5	4.0	2.0	1.0
Small bowel (1)	2.3	2.3	3.0	3.0	3.0
Ovaries (1)	2.7	1.3	4.0	2.0	1.0
Malignant peritoneal mesothelioma (1)	3.5	3.5	4.0	4.0	2.0
All patients (34)	2.9 (1.0)	2.1 (0.9)	3.3 (1.2)	2.6 (1.2)	2.4 (1.3)

QB-CM, clips marked quadrant biopsies; WB, visually most malignant features (worst biopsy); SD, standard deviation.

## Discussion

To our knowledge, this is the first study to evaluate the impact of different biopsy strategies on response evaluation using PRGS in patients with PM treated with PIPAC. This study showed that PRGS maximum from QB-CM was similar or significantly higher in most patients compared to PRGS from the PM element with visually most malignant features at the second PIPAC procedure (p<0.05). Thus, clips marking and reevaluation from the same area of the parietal peritoneum did not overestimate the histological treatment response.

The idea of clips marking PM during the first PIPAC was originally inspired by “target lesions” according to the RECIST criteria [6]. As such, it is possible to identify the worst lesions during the first PIPAC, and then follow them specifically. Further, it gives the opportunity to reduce sampling error, especially if different PIPAC surgeons treat each patient. It has been debated in international fora, if this strategy leads to false positive histological regression, since the scar tissue from the first biopsies may influence the histological interpretation. The data presented herein does not indicate such problem, since biopsies from QB-CM elements showed similar or poorer response to treatment compared to biopsies from WB in 30/34 (88%).

The PRGS was proposed in 2016 for histological response to therapy in PM, and it is recommended to take biopsies from suspect localizations from all four abdominal quadrants, but the re-biopsy strategy during subsequent PIPAC procedures is not specified [14]. The PRGS is widely adapted for histological response assessment during PIPAC directed treatment of patients with PM of different origin and is being used by the majority of PIPAC centers 50/62 (81%) [22]. According to a recent survey, 17/62 (27%) of the centers take biopsies from the same sites and 44/62 (71%) from alternate sites [22]. The number of PIPAC centers is increasing, and it is important to have a standardized implementation and practice that allows better data analysis and comparison of results. There is a high degree of consensus regarding safety and installation, but the least consensual topics are chemotherapy, and response evaluation [22].

Although PRGS has a moderate to good/substantial inter-observer variability and good to excellent/almost-perfect intra-observer variability for the assessment of response to treatment of PM, the prognostic impact of PRGS alone without cytology is not yet known [15]. One recent study showed that maximum PRGS combined with peritoneal cytology, had prognostic value on PIPAC treatment of PM [17]. Several ongoing studies use the PRGS as a main or secondary outcome, and these studies are expected to evaluate the prognostic impact of PRGS within a few years [18].

In the current study, we had one pathologist with interest in peritoneal pathology, who was blinded regarding the origin of the biopsies, which should be considered a strength. Patients with PM of any origin were included which is also considered a strength since it reflects the everyday patient treatment at a high volume PIPAC center. From a methodological point of view, it is a limitation due to the heterogenous study population regarding primary tumor origin, type of drugs used for PIPAC and different lines of systematic chemotherapy. Therefore, the findings of this study obviously need to be addressed in larger and more homogenous study populations. The accepted overlap between the WB and QB-CM could be considered both a weakness and a strength, but to avoid selection bias, it was pivotal for the independent surgical oncologist to be able to point out the WB, irrespective of clips, during the second PIPAC.

In conclusion, biopsies from clips marked quadrant biopsies did not overestimate treatment response compared to biopsies from the PM with visually most malignant features. Further studies are warranted, and they should investigate the optimal biopsy and response evaluation strategy in patients with PM.
